# Vascular Risk Factors and the Risk of Incident Parkinson’s Disease Among Stroke Survivors: A Nationwide Population-Based Cohort Study

**DOI:** 10.3390/jcm15166406

**Published:** 2026-08-19

**Authors:** Seo Yeon Yoon, Hyun Im Moon, Jin Hyung Jung, Sang Chul Lee, Kyungdo Han, Yong Wook Kim

**Affiliations:** 1 Department and Research Institute of Rehabilitation Medicine, Yonsei University College of Medicine, 50-1 Yonsei-ro Seodaemun-gu, Seoul 03722, Republic of Korea; syyoon23@yuhs.ac (S.Y.Y.); bettertomo@yuhs.ac (S.C.L.); 2 Department of Rehabilitation Medicine, Bundang Jesaeng General Hospital, Seongnam 13590, Republic of Korea; feellove99@gmail.com; 3 Samsung Biomedical Research Institute, Sungkyunkwan University School of Medicine, Suwon 16419, Republic of Korea; jungjin115@naver.com; 4 Department of Statistics and Actuarial Science, Soongsil University, 369 Sangdo-ro, Dongjak-gu, Seoul 06978, Republic of Korea

**Keywords:** stroke, Parkinson’s disease, vascular risk factor, cohort studies

## Abstract

**Background**: Stroke survivors often present vascular risk factors (VRFs) that are increasingly recognized as contributing to neurodegenerative diseases such as Parkinson’s disease (PD). We examined the influence of VRFs on the risk of PD in stroke survivors. **Methods**: Using the Korean National Health Insurance Service database, we identified 284,799 individuals with new-onset stroke and followed them for the occurrence of PD. The diagnosis of PD was confirmed using the International Classification of Diseases (ICD-10) code G20 and the rare disease registration code (V124). **Results**: Cox proportional hazard analyses revealed that smoking (former: Hazard ratio [HR] = 0.80; current: HR = 0.66) and alcohol consumption (moderate: HR = 0.83; heavy: HR = 0.74) were associated with a lower risk of PD. Conversely, diabetes mellitus, especially with a duration ≥ 5 years, was associated with increased PD risk (HR = 1.38). **Conclusions**: These findings suggest that several VRFs are associated with subsequent PD risk in stroke survivors. Further studies are warranted to determine whether modification of these risk factors influences the development of PD.

## 1. Introduction

Parkinson’s disease (PD) is a common and rapidly growing neurodegenerative disorder that is expected to double in prevalence by 2040 [1]. The clinical emergence of PD likely involves various risk factors, including both genetic and environmental factors that exert a long term influence on the onset of PD. However, the exact etiology of PD remains unclear [2,3].

Stroke is a common neurological disease among older adults and is the leading cause of long-term physical and cognitive disability. Managing vascular risk factors (VRFs) such as hypertension (HTN), diabetes mellitus (DM), and dyslipidemia is crucial for the primary prevention of stroke and secondary prevention of recurrent stroke [4]. High blood pressure (BP), glucose, and low-density lipoprotein cholesterol levels have also been associated with cognitive decline and dementia [5], whereas a recent study suggested that glucose levels were associated with faster cognitive decline among stroke survivors [6]. VRFs may accelerate cognitive decline through cerebral microvascular injury, oxidative stress, inflammation, and neurodegeneration [7,8,9]. These mechanisms have also been identified as potential contributors to the pathophysiology of PD in previous studies [10].

Identifying stroke survivors at increased risk of PD may facilitate individualized long-term follow-up and early recognition of parkinsonian symptoms, potentially improving patient care. Several studies have investigated the association between PD and stroke risk; however, the results have been inconsistent. Some studies have reported that the risk of stroke is significantly increased after a previous PD event [11,12,13], whereas others have reported a reduced risk of stroke in patients with PD [14], and some have not found any association between stroke and PD [15]. However, the risk of PD among stroke survivors has rarely been investigated [16]. A recent meta-analysis reported that PD and stroke may have a common pathogenesis and share preventive treatment measures [17]. Several biological mechanisms may underlie the association between VRFs and poststroke PD. Cerebral ischemia can induce persistent oxidative stress, neuroinflammation, endothelial dysfunction, and blood–brain barrier disruption, thereby promoting a neurodegenerative environment. Concurrent VRFs may exacerbate these processes through chronic vascular injury and metabolic dysregulation, potentially increasing the susceptibility of dopaminergic neurons to degeneration. These shared pathophysiological pathways provide the biological rationale for investigating the association between VRFs and PD among stroke survivors [2,18,19].

Recent nationwide cohort data demonstrated [16] that stroke survivors are at an increased risk of developing PD compared with the general population. Although numerous epidemiological studies have investigated the associations between individual vascular- and lifestyle-related risk factors, including smoking, alcohol consumption, DM, and dyslipidemia, and the risk of PD, the findings have been inconsistent. Moreover, these associations have rarely been evaluated specifically among stroke survivors, a population with a distinct vascular risk profile and a substantially increased risk of PD. To the best of our knowledge, no previous nationwide population-based cohort study has comprehensively evaluated the association between multiple VRFs and incidence of PD among stroke survivors. Identifying high-risk profiles after a stroke may provide opportunities for targeted prevention strategies and improve long-term neurological outcomes. Therefore, we conducted a nationwide population-based cohort study to comprehensively evaluate the association between multiple VRFs and the subsequent development of PD among stroke survivors.

## 2. Methods

### 2.1. Data Source

The Korean National Health Insurance Service (NHIS) provides mandatory health insurance coverage for all Koreans. All medical institutions electronically submit healthcare utilization data to the NHIS for reimbursement purposes, and these data are centralized in the NHIS database. The data contain a unique anonymized number for each individual and summarize demographic factors, type of insurance, diagnostic codes based on the International Classification of Diseases (ICD-10), procedures, medical costs claimed, and prescribed drugs. Additionally, the standardized National Health Screening Program (NHSP) is provided at least bi-annually to NHIS enrollees. The NHSP includes anthropometric measurements, BP assessment, laboratory investigations, and a self-reported questionnaire on lifestyle factors such as smoking, alcohol consumption, and physical activity.

### 2.2. Standard Protocol Approvals, Registrations, and Patient Consents

This study was approved by the Institutional Review Board of Yonsei University Severance Hospital (4-2023-1428) on 20 December 2023. The requirement for informed consent was waived by the Institutional Review Board of Yonsei University Severance Hospital because obtaining informed consent from all participants was impracticable and the study involved minimal risk to participants. All methods were performed in accordance with the relevant guidelines and regulations.

### 2.3. Study Population

**Figure 1:**
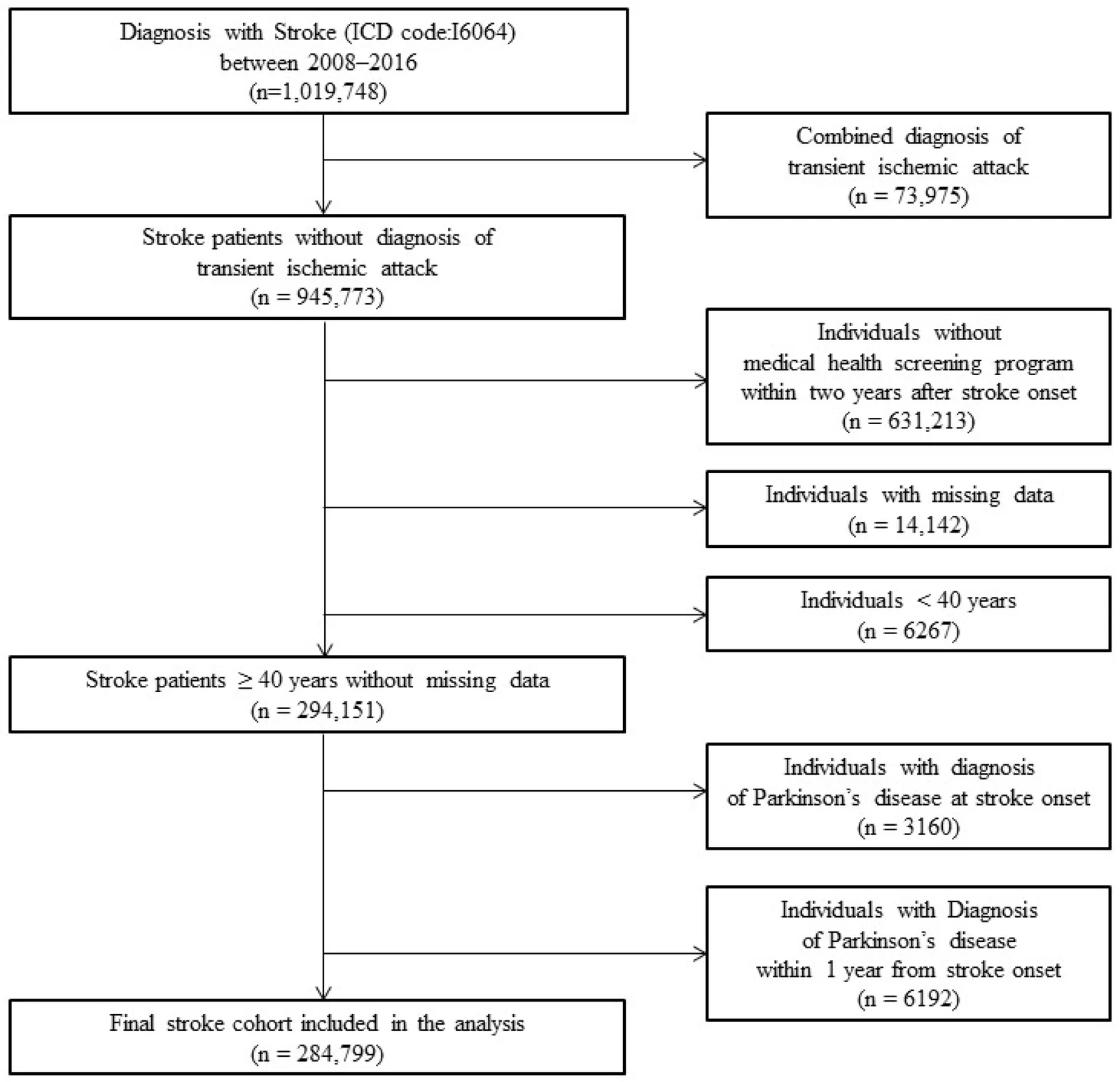
Flow diagram of participant selection and exclusions.

We included individuals newly diagnosed with stroke, defined by a primary diagnosis according to ICD-10 codes (I60–I64) between 2008 and 2016. Only individuals who were admitted to the hospital with a diagnosis of stroke based on brain imaging evaluations, such as magnetic resonance imaging or computed tomography, were included. Individuals with a concurrent diagnosis of transient ischemic attack were excluded from the study. Among the eligible participants, only those who underwent the NHSP within two years after their stroke diagnosis were included. Individuals aged <40 years, those with missing data, and those with a diagnosis of PD prior to enrollment were excluded. We set a 1-year lag period for PD diagnosis; therefore, individuals diagnosed with PD within one year of enrollment were also excluded. Finally, 284,799 individuals who had experienced a stroke were enrolled in the analysis and followed up until 31 December 2019 (Figure 1).

### 2.4. Definition of PD

The primary end point was newly diagnosed PD during the follow-up period. Since 2006, the Korean government has implemented a registration program for rare intractable diseases (RID), including PD, to assist patients with medical expenses. The diagnostic criteria for the V124 code are nearly identical to those of the UK Parkinson’s Disease Society Brain Bank criteria. Newly diagnosed PD was defined based on the ICD-10 code for PD (G20) and the RID registration code for PD (V124). In the sensitivity analysis, we excluded patients with a concurrent diagnosis of atypical or secondary parkinsonism (ICD-10 codes: G21-G23) to ensure a homogenized PD cohort.

### 2.5. Other Variables

Income status was approximated using insurance premium levels and classified into four groups (Q1–Q4) based on premium amount; low income was defined as the bottom 25%. Height, weight, waist circumference, and BP (systolic and diastolic) were assessed. The body mass index (BMI) was calculated as weight divided by height in meters squared (kg/m^2^). Venous blood samples were drawn after an overnight fast to determine fasting plasma glucose and total cholesterol (TC). Detailed information on health behaviors was obtained using standardized self-report questionnaires. The participants were classified according to their smoking status as never, former, or current smokers. Alcohol consumption was categorized as none, mild-to-moderate (<30 g/d), or heavy (≥30 g/d). Physically active individuals were defined as those who performed vigorous-intensity exercise three or more times per week or moderate-intensity exercise five or more times per week. Baseline comorbidities (DM, HTN, and dyslipidemia) and their statuses were identified based on a combination of ICD-10 codes, laboratory, and anthropometric findings on the NHSP, and data from the prescription database. DM was classified based on glycemic status as no DM (fasting glucose < 100 mg/dL), impaired fasting glucose (fasting glucose 100–125 mg/dL), incident DM (fasting glucose ≥ 126 mg/dL without DM medication), DM duration < 5 years, and DM duration ≥ 5 years. HTN was classified based on BP as no HTN (SBP < 120 mmHg and DBP < 80 mmHg), pre-HTN (SBP 120–139 mmHg or DBP 80–89 mmHg), incident HTN (SBP ≥ 140 mmHg or DBP ≥ 90 mmHg without HTN medication), controlled HTN (SBP < 140 mmHg and DBP < 90 mmHg with HTN medication), and uncontrolled HTN (SBP ≥ 140 mmHg or DBP ≥ 90 mmHg with HTN medication). Dyslipidemia was classified based on TC level as no dyslipidemia (TC < 200 mg/dL), pre-dyslipidemia (TC 200–239 mg/dL), incident dyslipidemia (TC ≥ 240 mg/dL without dyslipidemia medication), controlled dyslipidemia (TC < 240 mg/dL with dyslipidemia medication), and uncontrolled dyslipidemia (TC ≥ 240 mg/dL with dyslipidemia medication).

### 2.6. Statistical Analysis

Baseline characteristics of stroke survivors are presented as the mean ± standard deviation for continuous variables or as the number (percentage) for categorical variables. Baseline differences across stroke types were evaluated using standardized mean differences rather than statistical testing because the large sample size rendered trivial differences statistically significant; an SMD > 0.10 was considered to indicate a meaningful between-group difference. The incidence of PD was calculated by dividing the number of events by 1000 person-years. Follow-up began on the date of the health screening examination. We conducted Cox proportional hazards analyses to evaluate the association between various risk factors, including socioeconomic status, lifestyle factors, and comorbidities, with the incidence of PD in stroke survivors, and estimated the hazard ratio (HR) and 95% confidence interval (CI). Death during follow-up was treated as censoring and cause-specific HRs were estimated. Model 1 was unadjusted. Model 2 was adjusted for age, sex, income, lifestyle factors, BMI, and comorbidities. Model 3 was further adjusted for mental disorders and antiplatelet drug use. Sensitivity analysis was performed after excluding the concurrent diagnosis of atypical or secondary parkinsonism from the PD group to ensure homogeneity. Stratified analyses were performed according to sex and type of stroke. The proportional hazard assumption was assessed using Schoenfeld residuals and log-minus-log survival plots. All statistical analyses were performed using SAS 9.4 (SAS Institute Inc., Cary, NC, USA), and a two-sided *p* < 0.05 was considered statistically significant.

## 3. Results

### 3.1. Characteristics of the Study Population

Table 1 presents the characteristics of the 284,799 stroke survivors according to the stroke type. Among the study population, 225,513 (79.2%), 46,007 (16.2%), and 13,279 (4.7%) individuals had ischemic, hemorrhagic, and unspecified stroke types, respectively. The ischemic stroke group had a higher proportion of men, and the mean age at stroke onset was approximately 6 years higher than that of the hemorrhagic stroke group. Individuals with ischemic stroke had a higher prevalence of DM, HTN, and dyslipidemia than those with hemorrhagic stroke. During follow-up, PD developed in 2826 (1.25%) individuals with ischemic stroke, 289 (0.63%) with hemorrhagic stroke, and 159 (1.2%) with unspecified stroke.

### 3.2. Risk Factors for PD Occurrence in Stroke Survivors

Table 2 presents various risk factors for PD occurrence. During the follow-up period (median 5.04 years, interquartile range 2.96–7.28), 3274 cases of PD (1.15%) occurred in individuals with stroke. Male sex (HR = 1.42, 95% CI, 1.30–1.54) and older age (HR = 3.28, 95% CI, 3.00–3.58) were significantly associated with an increased risk of PD. Among individuals with a stroke, former (HR = 0.80, 95% CI, 0.72–0.88) and current (HR = 0.66, 95% CI, 0.58–0.76) smoking status showed a significant association with reduced risk of PD. Alcohol consumption was also associated with a decreased risk of PD in individuals with stroke (moderate drinking, HR = 0.83, 95% CI, 0.75–0.92; heavy drinking, HR = 0.74, 95% CI, 0.59–0.94). The association between physical activity and PD occurrence did not reach statistical significance (*p* = 0.0508) (HR = 0.91, 95% CI, 0.83–1.00, *p* = 0.0508). As for the association between comorbidities and PD risk, the risk of PD increased among stroke survivors with DM (*p* for trend <0.001), particularly among those with a DM duration ≥ 5 years, in whom the PD risk increased by 38% (HR = 1.38, 95% CI, 1.25–1.52). The PD risk was reduced in patients who had a stroke with dyslipidemia (*p* for trend = 0.0003), whereas BMI and HTN were not significantly associated with PD risk in stroke survivors (*p* > 0.05).

The sensitivity analysis, which excluded concurrent diagnosis of secondary or atypical parkinsonism from the PD diagnosis, yielded results similar to the main results (Supplementary Table S1).

### 3.3. Subgroup Analysis by Sex and Stroke Type

Table 3 presents the association of various factors with PD risk in individuals with a history of stroke, stratified by sex. Differences in the association patterns of smoking and dyslipidemia with PD risk between males and females were observed. Smoking (former, HR = 0.80, 95% CI, 0.78–0.89; current, HR = 0.66, 95% CI, 0.57–0.77, *p* for trend <0.0001) and dyslipidemia (incident dyslipidemia, HR = 0.64, 95% CI, 0.47–0.86; controlled dyslipidemia, HR = 0.82, 95% CI, 0.73–0.92, *p* for trend = 0.0008) were significantly associated with a reduced PD risk only in males. For alcohol consumption, moderate alcohol consumption showed a numerically stronger inverse association with PD risk in females (HR = 0.51, 95% CI 0.37–0.70) than in males (HR = 0.90, 95% CI 0.80–1.01). DM was significantly associated with PD risk in both males (*p* = 0.0111) and females (*p* <0.0001).

**Figure 2:**
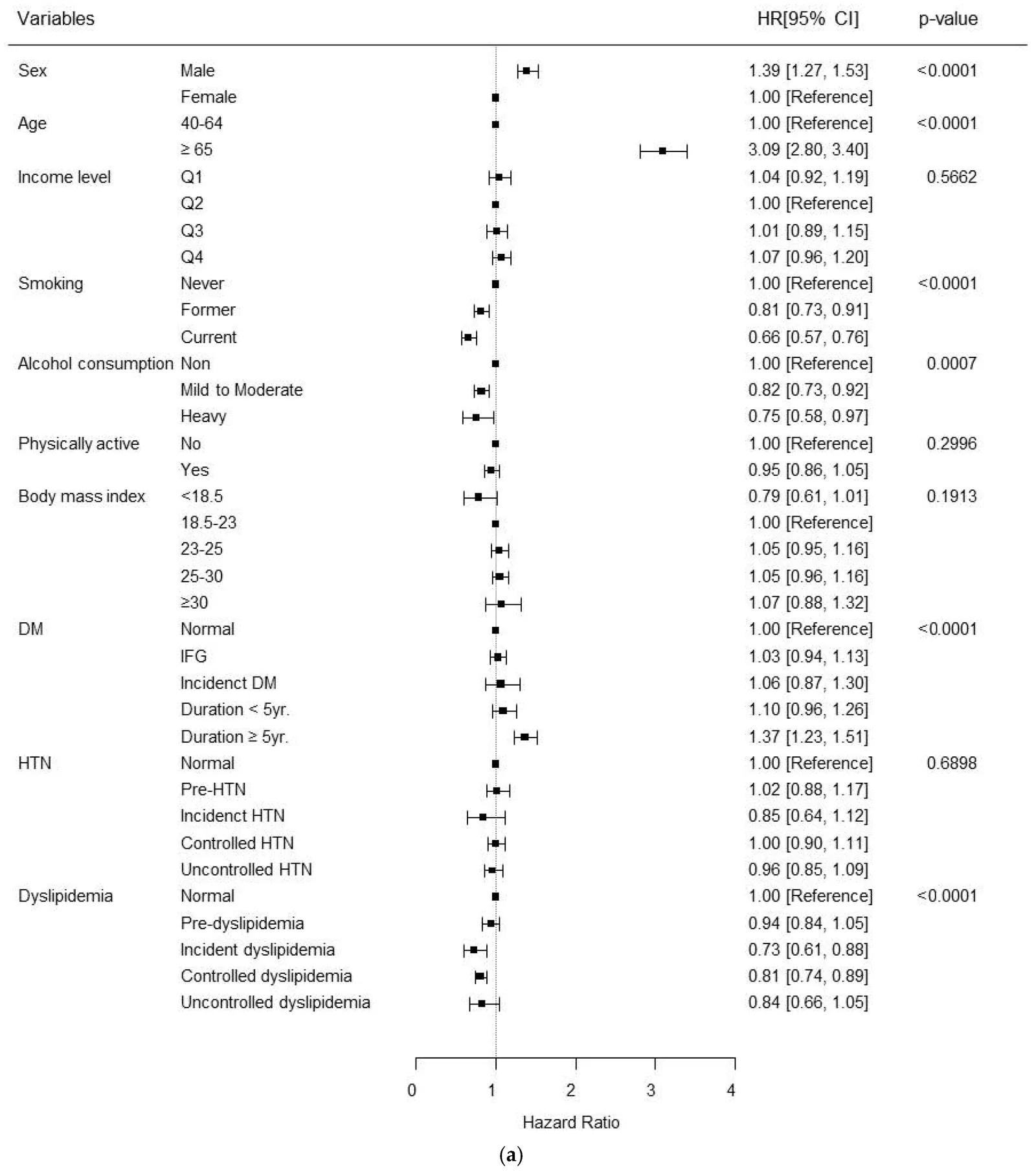

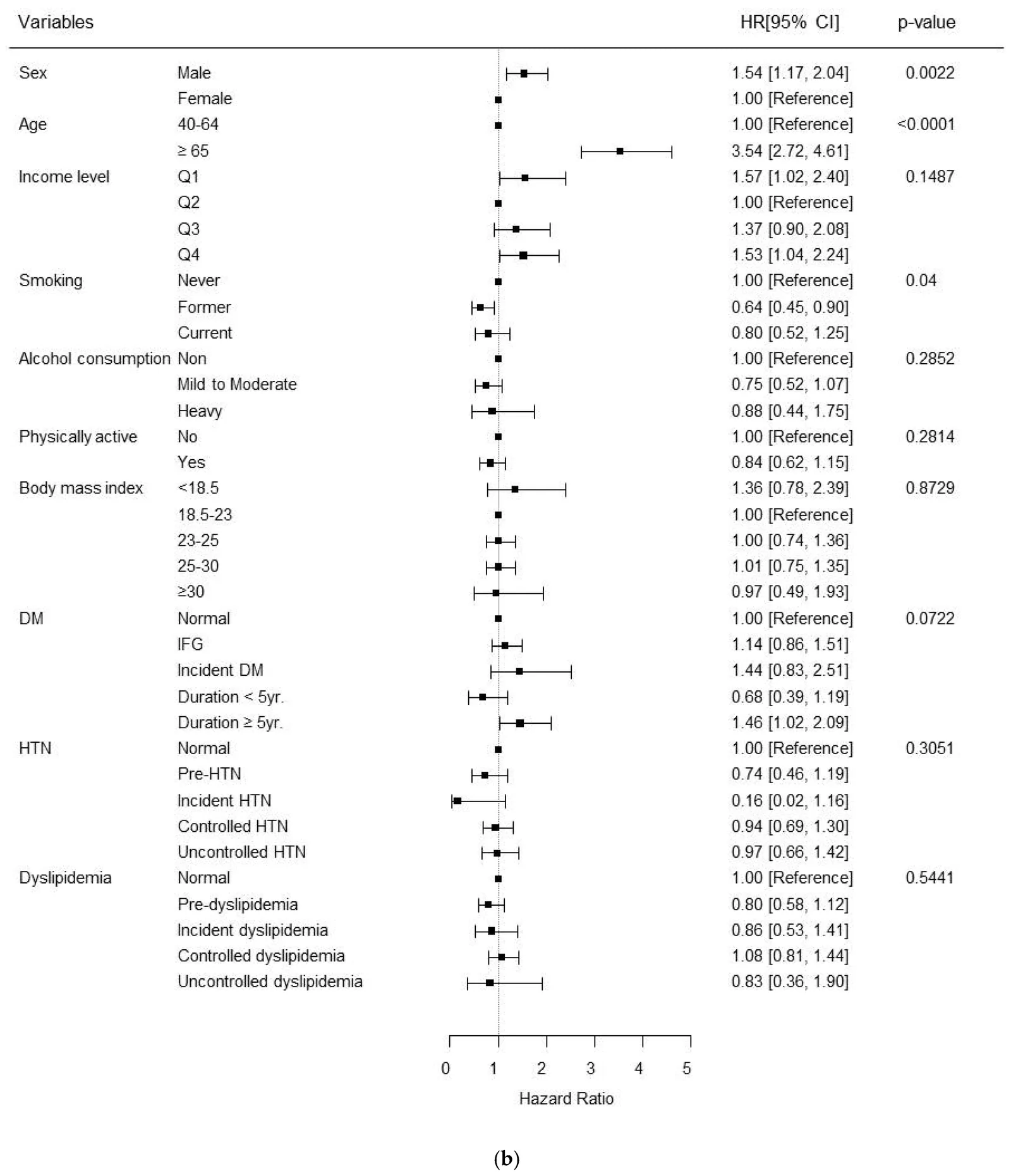
Subgroup analysis of PD risk by stroke type and sex. (**a**) Ischemic stroke and (**b**) hemorrhagic stroke.

The association between various factors and PD risk in stroke survivors, stratified by stroke type, is presented in Figure 2. Overall, smoking (*p* for trend <0.0001), alcohol consumption (*p* for trend = 0.0007), dyslipidemia (*p* for trend <0.0001), and DM (*p* for trend <0.0001) were significantly associated with PD in individuals with ischemic stroke than but not among those with hemorrhagic stroke.

## 4. Discussion

In this nationwide cohort of 284,799 stroke survivors, we identified several VRFs associated with the subsequent development of PD. Although previous studies have primarily focused on whether stroke itself increases the risk of PD, our findings suggest that the risk of PD among stroke survivors is not uniform and may vary according to modifiable vascular and lifestyle-related factors, particularly DM, dyslipidemia, smoking status, and alcohol consumption.

The absolute number of individuals affected by stroke (i.e., those who die from or remain disabled due to stroke) has almost doubled over the past three decades, with >86% of the stroke burden occurring in low-and middle-income countries [20]. Therefore, many stroke survivors live with restrictions in cognition and physical function as sequelae. VRFs are considered important for secondary prevention in stroke survivors, and previous studies have reported associations between VRFs and cognitive decline after stroke [6,21]. Patients with stroke often present with physical dysfunction, and VRFs may cause motor dysfunction, such as vascular parkinsonism, thereby further worsening physical function. PD is the second most common neurodegenerative disease that occurs frequently in old age, and studies on the association between VRFs and PD occurrence have recently been published [15,22,23]. Considering that PD occurrence in patients with stroke can worsen their functional level and long-term prognosis, our study analyzed the relationship between VRFs and PD occurrence. Our findings suggest that various VRFs may influence the risk of PD among stroke survivors, highlighting the importance of managing VRFs for physical function among stroke survivors.

In our study, old age and male sex were significant risk factors for PD among stroke survivors, which is consistent with previous studies conducted in the general population [24,25]. Regarding the association between comorbidities and PD risk, DM was significantly associated with an increased risk of PD among stroke survivors, particularly in those with a DM duration of ≥5 years. Recent evidence has increasingly supported the association between DM and PD, with several systematic reviews and meta-analyses reporting a significantly increased risk of PD in individuals with DM [26,27,28]. Proposed mechanisms include insulin resistance, mitochondrial dysfunction, oxidative stress, neuroinflammation, and impaired proteostasis, all of which may contribute to dopaminergic neuronal degeneration and α-synuclein aggregation [26]. Furthermore, a longer DM duration may reflect a greater cumulative metabolic burden, potentially accelerating neurodegenerative processes [26,27]. Our findings are consistent with these observations and suggest that chronic metabolic dysfunction may play an important role in the development of PD among stroke survivors.

Regarding dyslipidemia, our findings indicate that stroke patients with dyslipidemia have a reduced risk of PD. However, the relationship between dyslipidemia and PD remains unclear. Some studies have suggested that statins may lower the incidence of PD, possibly owing to their anti-inflammatory and neuroprotective properties [29,30]. However, other studies have reported a negative association between lipid levels and PD, with evidence showing that elevated cholesterol levels may be linked to a lower risk of PD in populations not taking statins [31,32]. Recent evidence has further highlighted the complex role of lipid metabolism in neurodegeneration, suggesting that cholesterol homeostasis may influence α-synuclein aggregation, neuronal membrane integrity, and dopaminergic signaling [26]. In this context, our classification incorporated both lipid levels and medication history. The inverse association was most pronounced in the untreated group, suggesting that statin use is not the sole explanation. Nevertheless, our definition did not capture the specific agent, dose, duration, or adherence, and therefore could not fully disentangle the effects of lipid levels from the pharmacological actions of lipid-lowering therapies. Accordingly, these findings should not be interpreted as evidence that dyslipidemia itself is protective against PD. Further studies incorporating longitudinal lipid profiles and cumulative medication exposure are warranted to clarify these relationships. Therefore, the observed association between dyslipidemia and reduced PD risk should be interpreted with caution and warrants further investigation.

In our study, no significant associations between BMI or HTN and the risk of PD were observed among stroke survivors. Few studies have focused on the effect of a previous HTN diagnosis on the risk of PD; some studies have yielded a reduced risk of PD, whereas others have shown no effect [33,34,35]. Additionally, a recent meta-analysis showed that patients with PD had a significantly lower BMI than controls, and some studies have shown an inverse association between BMI and PD [36]. Among the VRFs mentioned above, DM and dyslipidemia appear to be more strongly associated with PD occurrence. A previous report proposed a specific interaction between VRFs and PD, which involves accelerating the neurodegenerative process, particularly in the presence of DM [37].

In the present study, former and current smoking were associated with a lower risk of developing PD. However, this finding appears to present a dilemma. Patients who have had a stroke should quit smoking to prevent recurrent strokes; therefore, smoking cessation is usually recommended. Nonetheless, regarding the association between smoking and PD, many studies have supported a reduced risk of PD among smokers, which is in line with our findings. A previous meta-analysis reported that smokers have a lower risk of PD [38]. Another epidemiological study reported that ever having smoked a cigarette was associated with a reduced risk of PD [39]. Alcohol consumption was also associated with a decreased risk of PD in individuals who had experienced a stroke. The effects of alcohol consumption on disease, in both PD and stroke, remain controversial. Studies have shown that moderate drinking has been associated with a reduced risk of ischemic stroke by increasing high-density lipoprotein cholesterol levels and suppressing blood clotting. However, excessive drinking can increase the risk of hemorrhagic and ischemic stroke. Alcohol abuse can worsen stroke risk factors such as high BP, heart arrhythmia, and atrial fibrillation [40,41]. Likewise, the association between alcohol consumption and the development of PD remains unclear, although research results are inconsistent. Some studies have suggested that moderate drinking may slightly reduce the risk of PD, but others have shown no clear relationship or that drinking does not significantly affect the risk of PD [38,39,42]. The possible mechanism for the neuroprotective effect of moderate drinking is that the antioxidants contained in alcoholic beverages, such as polyphenols, can protect dopaminergic neurons [43]. However, inverse associations between smoking, alcohol consumption, and PD should be interpreted with caution because of possible reverse causation. Given the long prodromal phase of PD, subclinical disease may lead to reduced smoking or alcohol intake years before diagnosis. Although we applied a 1-year lag period to mitigate this, the impact of a longer prodromal window cannot be fully excluded.

Physical activity was not significantly associated with PD risk. Physical activity or exercise is recommended for stroke survivors to reduce the risk of recurrent stroke, and evidence suggests that physical activity reduces the risk of PD [44]. Stroke survivors often have functional limitations that may restrict their ability to perform moderate or vigorous physical activity, potentially influencing our results.

In the subgroup analysis according to sex, smoking and dyslipidemia were significantly associated with a lower PD risk only in males. These sex-specific differences likely reflected the combined effects of biological and epidemiological factors. For smoking, the prevalence of smoking among Korean women is extremely low, which may have limited the ability to draw statistically meaningful results. A similar explanation may apply to alcohol consumption. The low prevalence and cumulative intensity of smoking and alcohol use among Korean women narrow the exposure contrast available to detect associations. Regarding dyslipidemia, the complex interaction between sex and lipid metabolism in relation to PD is unclear. However, the modulatory effects of sex hormones, such as estrogen and androgen, on lipoprotein metabolism should be considered [45]. Estrogen may additionally confer neuroprotection through antioxidant and anti-inflammatory mechanisms, as well as modulation of nigrostriatal dopaminergic function; such baseline protection could plausibly attenuate the incremental contribution of VRFs in women [46,47]. Taken together, these biological and exposure-related differences may jointly account for the heterogeneity observed across sexes, although this interpretation remains speculative. Considering that previous reports have shown that the occurrence and clinical characteristics of PD differ according to sex, further studies are needed to confirm these sex-specific characteristics. In the subgroup analysis according to stroke type, smoking, alcohol consumption, dyslipidemia, and DM showed a more significant association with PD in patients with ischemic stroke than in those with hemorrhagic stroke. This may be explained by the characteristics of the study population. In our study, the prevalence of obesity, DM, HTN, and dyslipidemia was higher in the ischemic stroke group than in the hemorrhagic stroke group. Our findings suggest that VRFs require more attention and should be appropriately controlled in relation to PD risk, especially in ischemic stroke survivors.

This study had some limitations that should be acknowledged. First, we used claims-based data, and misclassification of PD and stroke cannot be excluded. Although the V124 registration process applies standardized diagnostic criteria and physician certification, the positive predictive value of the combined G20 and V124 definition has not, to our knowledge, been directly validated against medical-record review in the Korean NHIS database. We used a strict operational definition of PD and performed a sensitivity analysis, excluding the concurrent diagnosis of atypical or secondary parkinsonism to ensure homogeneity. Nevertheless, idiopathic PD cannot be perfectly distinguished from vascular parkinsonism because poststroke functional impairment may obscure the clinical differentiation between these conditions. Moreover, excluding secondary parkinsonism codes (G21–G23) may not completely eliminate this possibility. Consequently, part of the observed association between VRFs, especially DM, and incident PD may reflect misclassified vascular parkinsonism. In addition, stroke severity, lesion location (particularly involvement of the basal ganglia or the nigrostriatal pathways), and recurrent stroke were unavailable in our database. These factors may influence the development of parkinsonian syndromes and may also correlate with VRFs; therefore, residual confounding cannot be excluded.

Second, our study included only stroke survivors who underwent NHSP within two years after stroke. Consequently, patients with severe disability, critical illness, or poor functional status who were unable to participate in health screening may have been underrepresented, and caution is warranted when generalizing our findings to the overall population of stroke survivors. Detailed clinical information regarding stroke severity, including National Institutes of Health Stroke Scale score, modified Rankin Scale score, infarct volume, and other neuroimaging findings, was unavailable. Because stroke severity may influence long-term functional outcomes, healthcare utilization, medical surveillance, and the likelihood of subsequent PD diagnosis, residual confounding related to unavailable clinical variables cannot be completely excluded.

Third, a surveillance bias may have influenced the observed associations. Because registration under the V124 program requires specialist evaluation and administrative enrollment, differences in healthcare access may have affected the detection of PD. Individuals with higher socioeconomic status may have been more likely to receive a diagnosis and V124 registration, potentially contributing to the higher observed incidence in the highest-income group. Similarly, patients with DM or multiple comorbidities may have more frequent healthcare encounters, increasing opportunities for PD diagnosis, whereas individuals with greater poststroke disability may undergo fewer neurological evaluations, potentially leading to underdiagnosis. In addition, because death during follow-up was treated as censoring rather than being modeled using a formal competing-risk approach, such as the Fine–Gray subdistribution hazard model, competing mortality among stroke survivors may have influenced the estimated cumulative incidence of PD. However, because the primary objective of this study was to evaluate the etiological associations between VRFs and incident PD rather than to estimate the absolute cumulative incidence, the cause-specific Cox proportional hazards model remains an appropriate analytical approach for addressing this research question. Therefore, the observed associations should be interpreted with caution.

Fourth, lifestyle factors were assessed using self-reported questionnaires and categorized using relatively simple classifications without quantitative cumulative exposure, such as smoking pack-years, lifetime alcohol consumption, or detailed measures of physical activity; therefore, misclassification and recall bias cannot be excluded. Because lifelong abstainers could not be distinguished from former drinkers, the observed association with alcohol consumption may also be partly influenced by the “sick quitter” effect. Dietary factors such as caffeine consumption were unavailable in the database. Furthermore, although DM, hypertension, and dyslipidemia were classified using NHSP measurements, diagnostic codes, and prescription records, detailed information on long-term metabolic control (e.g., HbA1c levels), cumulative disease burden, lipid subfractions, and medication exposure, including the duration and intensity of statin therapy and other specific antidiabetic and lipid-lowering agents, was not evaluated; therefore, residual confounding related to pharmacological treatment cannot be excluded.

Finally, VRFs were assessed only once at baseline health screening after stroke; changes in lifestyle habits and metabolic status during follow-up could not be captured, potentially resulting in exposure misclassification and attenuation of the observed associations. In addition, because the follow-up duration varied according to the year of stroke onset, secular changes in diagnostic or surveillance practices over the enrollment period may have influenced the long-term estimates.

Because patients who had a stroke generally have a higher burden of VRFs than the general population, lifestyle interventions, including attention to controlling VRFs, especially DM, should be encouraged as part of a comprehensive approach to the management of patients newly diagnosed with stroke to reduce the risk of PD.

## 5. Conclusions

In this nationwide cohort of stroke survivors, DM was independently associated with an increased risk of subsequent PD, whereas smoking, alcohol consumption, and dyslipidemia were associated with a lower risk of PD. Because of the observational design, these findings should be interpreted as associations rather than as evidence of causal or protective effects. Further studies are needed to determine whether modification of VRFs influences the development of PD after stroke.

## Figures and Tables

**Table 1:** Characteristics of individuals with stroke.

	Stroke Type			
	Ischemic (n = 225,513)	Hemorrhagic (n = 46,007)	Unspecified (n = 13,279)	SMD
Male	121,854 (54.03)	24,358 (52.94)	6260 (47.14)	0.138
Age (years)	66.26 ± 10.47	60.5 ± 11.16	63.93 ± 11.12	0.532
Low-income level	45,925 (20.36)	9672 (21.02)	2788 (21)	0.016
Smoking				0.111
Never	144,798 (64.21)	29,301 (63.69)	8994 (67.73)	
Former	49,993 (22.17)	10,845 (23.57)	2527 (19.03)	
Current	30,722 (13.62)	5861 (12.74)	1758 (13.24)	
Alcohol consumption				0.090
None	174,198 (77.25)	34,532 (75.06)	9754 (73.45)	
Moderate	43,195 (19.15)	9520 (20.69)	2915 (21.95)	
Heavy	8120 (3.6)	1955 (4.25)	610 (4.59)	
Physically active	43,367 (19.23)	9460 (20.56)	2601 (19.59)	0.033
Body mass index (kg/m^2^)				0.091
<18.5	6469 (2.87)	1488 (3.23)	361 (2.72)	
18.5–23	71,661 (31.78)	16,036 (34.86)	4143 (31.2)	
23–25	59,279 (26.29)	11,787 (25.62)	3504 (26.39)	
25–30	78,837 (34.96)	14,904 (32.4)	4699 (35.39)	
≥30	9267 (4.11)	1792 (3.9)	572 (4.31)	
DM				0.283
Normal	104,141 (46.18)	25,665 (55.78)	6726 (50.65)	
IFG	55,937 (24.8)	11,906 (25.88)	3466 (26.1)	
Incident DM	8760 (3.88)	1703 (3.7)	553 (4.16)	
Duration < 5 yr.	20,712 (9.18)	2998 (6.52)	827 (6.23)	
Duration ≥ 5 yr.	35,963 (15.95)	3735 (8.12)	1707 (12.85)	
HTN				0.250
Normal	44,820 (19.87)	9757 (21.21)	3825 (28.8)	
Pre-HTN	26,472 (11.74)	5704 (12.4)	1930 (14.53)	
Incident HTN	5413 (2.4)	1036 (2.25)	308 (2.32)	
Controlled HTN	102,857 (45.61)	21,731 (47.23)	5070 (38.18)	
Uncontrolled HTN	45,951 (20.38)	7779 (16.91)	2146 (16.16)	
Dyslipidemia				0.553
Normal	59,197 (26.25)	19,204 (41.74)	4312 (32.47)	
Pre-dyslipidemia	32,939 (14.61)	10,254 (22.29)	2845 (21.42)	
Incident dyslipidemia	11,843 (5.25)	3711 (8.07)	958 (7.21)	
Controlled dyslipidemia	115,063 (51.02)	11,792 (25.63)	4750 (35.77)	
Uncontrolled dyslipidemia	6471 (2.87)	1046 (2.27)	414(3.12)	
Wasit circumference (cm)	83.79 ± 8.59	82.61 ± 8.81	83.19 ± 8.74	0.136
Systolic BP (mmHg)	128.35 ± 15.84	126.17 ± 15.31	126.47 ± 15.35	0.140
Diastolic BP (mmHg)	77.79 ± 10.14	78.07 ± 10.2	77 ± 9.87	0.107
Fasting glucose (mg/dL)	107.34 ± 31.96	101.89 ± 26.18	105.01 ± 29.68	0.187
Total cholesterol (mg/dL)	178.75 ± 42.09	189.05 ± 40.29	188.9 ± 40.45	0.25
GFR	81.78 ± 40.03	87.3 ± 41.58	83.42 ± 37.98	0.135

Values are presented as mean ± SD or number (%). SMD, standardized mean difference; DM, diabetes mellitus; IFG, impaired fasting glucose; HTN, hypertension; BP, blood pressure.

**Table 2:** Cox proportional hazard regression analysis of the Parkinson’s Disease risk in individuals with stroke.

		N	PD	Person–Years	Incidence Rate	Model 1	*p*	Model 2	*p*	Model 3	*p*
Sex	Male	152,472	1699	758,466.98	2.24	1.00 (0.93–1.07)		1.34 (1.23–1.46)		1.42 (1.30–1.54)	
	Female	132,327	1575	704,011.1	2.24	1.00	0.9158	1.00	<0.0001	1.00	<0.0001
Age (years)	40–64	131,539	673	713,849.71	0.94	1.00	<0.0001	1.00	<0.0001	1.00	<0.0001
	≥65	153,260	2601	748,628.37	3.47	3.67 (3.37–3.99)		3.37 (3.08–3.68)		3.28 (3.00–3.58)	
Income level	Q1	58,385	613	292,544.94	2.10	1.14 (1.01–1.28)		1.11 (0.98–1.25)		1.10 (0.98–1.24)	
	Q2	48,438	459	249,396.56	1.84	1.00	<0.0001	1.00	0.0946	1.00	0.1184
	Q3	68,630	744	355,985.81	2.09	1.14 (1.01–1.28)		1.05 (0.93–1.18)		1.05 (0.93–1.18)	
	Q4	109,346	1458	564,550.77	2.58	1.40 (1.26–1.56)		1.13 (1.02–1.25)		1.13 (1.01–1.25)	
Smoking	Never	183,093	2382	954,014.59	2.50	1.00	<0.0001	1.00	<0.0001	1.00	<0.0001
	Former	63,365	623	313,030.67	1.99	0.79 (0.73–0.87)		0.80 (0.72–0.88)		0.80 (0.72–0.88)	
	Current	38,341	269	195,432.82	1.38	0.55 (0.49–0.62)		0.67 (0.58–0.77)		0.66 (0.58–0.76)	
Alcohol consumption	None	218,484	2741	1,114,262.02	2.46	1.00	<0.0001	1.00	<0.0001	1.00	0.0003
	Moderate	55,630	458	291,815.14	1.57	0.64 (0.58–0.71)		0.80 (0.72–0.89)		0.83 (0.75–0.92)	
	Heavy	10,685	75	56,400.92	1.33	0.54 (0.43–0.68)		0.71 (0.57–0.90)		0.74 (0.59–0.94)	
Physically active	No	229,371	2711	1,171,360.73	2.31	1.00	0.0001	1.00	0.0343	1.00	0.0508
	Yes	55,428	563	291,117.35	1.93	0.84 (0.76–0.92)		0.91 (0.83–0.99)		0.91 (0.83–1.00)	
Body mass index	<18.5	8318	79	35,387.35	2.23	0.97 (0.77–1.22)		0.85 (0.68–1.07)		0.84 (0.66–1.05)	
	18.5–23	91,840	1051	461,942.06	2.28	1.00	0.6946	1.00	0.379	1.00	0.2205
	23–25	74,570	884	388,966.49	2.27	1.00 (0.92–1.10)		1.04 (0.95–1.14)		1.05 (0.96–1.15)	
	25–30	98,440	1142	516,982.27	2.21	0.97 (0.90–1.06)		1.05 (0.97–1.15)		1.07 (0.98–1.16)	
	≥30	11,631	118	59,199.9	1.99	0.88 (0.73–1.06)		1.03 (0.85–1.24)		1.04 (0.86–1.26)	
DM	Normal	136,532	1463	724,839.74	2.02	1.00	<0.0001	1.00	<0.0001	1.00	<0.0001
	IFG	71,309	781	363,161.31	2.15	1.06 (0.97–1.16)		1.04 (0.95–1.13)		1.03 (0.95–1.13)	
	Incident DM	11,016	126	55,115.6	2.29	1.13 (0.94–1.36)		1.11 (0.93–1.34)		1.12 (0.93–1.34)	
	Duration < 5 yr.	24,537	276	127,357	2.17	1.07 (0.94–1.22)		1.04 (0.91–1.19)		1.04 (0.91–1.19)	
	Duration ≥ 5 yr.	41,405	628	192,004.43	3.27	1.61 (1.46–1.76)		1.39 (1.26–1.53)		1.38 (1.25–1.52)	
HTN	Normal	58,402	575	301,708.41	1.91	1.00	<0.0001	1.00	0.2879	1.00	0.3768
	Pre–HTN	34,106	361	180,576.01	2.00	1.05 (0.92–1.20)		1.03 (0.90–1.18)		1.04 (0.91–1.18)	
	Incident HTN	6757	59	36,686.7	1.61	0.85 (0.65–1.11)		0.78 (0.60–1.02)		0.80 (0.61–1.04)	
	Controlled HTN	129,658	1574	656,763.83	2.40	1.26 (1.14–1.38)		1.01 (0.92–1.12)		0.99 (0.90–1.09)	
	Uncontrolled HTN	55,876	705	286,743.14	2.46	1.29 (1.16–1.44)		0.97 (0.86–1.08)		0.96 (0.86–1.08)	
Dyslipidemia	Normal	82,713	1078	446,979.73	2.41	1.00	0.0005	1.00	0.0012	1.00	0.0003
	Pre– dyslipidemia	46,038	545	257,968.12	2.11	0.88 (0.79–0.97)		0.93 (0.83–1.03)		0.93 (0.83–1.03)	
	Incident dyslipidemia	16,512	158	93,203.06	1.70	0.71 (0.60–0.83)		0.75 (0.63–0.89)		0.75 (0.64–0.89)	
	Controlled dyslipidemia	131,605	1402	621,024.33	2.26	0.93 (0.85–1.00)		0.87 (0.80–0.94)		0.85 (0.78–0.92)	
	Uncontrolled dyslipidemia	7931	91	43,302.85	2.10	0.87 (0.70–1.08)		0.91 (0.73–1.13)		0.86 (0.69–1.06)	

DM, diabetes mellitus; IFG, impaired fasting glucose; HTN, hypertension. The incidence rate was defined as the incidence of Parkinson’s disease per 1000 person-years. Model 1: unadjusted. Model 2: Adjusted for age, sex, income level, lifestyle factors, body mass index, DM, HTN, and dyslipidemia. Model 3: Adjusted for age, sex, income level, lifestyle factors, body mass index, DM, HTN, dyslipidemia, mental disorders, and antiplatelet use.

**Table 3:** Subgroup analysis of the Parkinson’s Disease Risk in Individuals with Stroke according to Sex.

		Male	*p* for Trend	Female	*p* for Trend
Age	40–64	1.00	<0.0001	1.00	<0.0001
	≥65	3.27 (2.90–3.68)		3.20 (2.80–3.67)	
Income level	Q1	1.06 (0.89–1.25)		1.15 (0.97–1.37)	
	Q2	1.00	0.071	1.00	0.4566
	Q3	1.02 (0.87–1.20)		1.08 (0.92–1.28)	
	Q4	1.16 (1.01–1.35)		1.08 (0.93–1.26)	
Smoking	Never	1.00	<0.0001	1.00	0.0551
	Former	0.80 (0.78–0.89)		0.66 (0.39–1.09)	
	Current	0.66 (0.57–0.77)		0.67 (0.43–1.04)	
Alcohol consumption	None	1.00	0.0184	1.00	0.0001
	Moderate	0.90 (0.80–1.01)		0.51 (0.37–0.70)	
	Heavy	0.75 (0.59–0.95)		1.20 (0.39–3.76)	
Physically active	No	1.00	0.2248	1.00	0.1108
	Yes	0.93 (0.83–1.05)		0.88 (0.76–1.03)	
Body mass index (kg/m^2^)	<18.5	0.93 (0.68–1.28)		0.75 (0.53–1.04)	
	18.5–23	1.00	0.8218	1.00	0.3095
	23–25	1.04 (0.92–1.18)		1.06 (0.92–1.20)	
	25–30	1.07 (0.95–1.20)		1.06 (0.94–1.20)	
	≥30	1.02 (0.73–1.41)		1.04 (0.82–1.32)	
DM	Normal	1.00	0.0111	1.00	.0001
	IFG	0.98 (0.87–1.11)		1.09 (0.96–1.24)	
	Incident DM	1.10 (0.86–1.39)		1.13 (0.85–1.49)	
	Duration < 5 yr.	0.92 (0.77–1.11)		1.18 (0.98–1.43)	
	Duration ≥ 5 yr.	1.23 (1.08–1.41)		1.55 (1.35–1.78)	
HTN	Normal	1.00	0.455	1.00	0.5987
	Pre-HTN	1.01 (0.84–1.20)		1.07 (0.88–1.30)	
	Incident HTN	0.73 (0.50–1.06)		0.87 (0.60–1.27)	
	Controlled HTN	1.03 (0.90–1.18)		0.95 (0.82–1.10)	
	Uncontrolled HTN	0.98 (0.83–1.15)		0.93 (0.79–1.10)	
Dyslipidemia	Normal	1.00	0.0008	1.00	0.1507
	Pre-dyslipidemia	0.91 (0.79–1.05)		0.95 (0.82–1.11)	
	Incident dyslipidemia	0.64 (0.47–0.86)		0.83 (0.68–1.03)	
	Controlled dyslipidemia	0.82 (0.73–0.92)		0.89 (0.78–1.01)	
	Uncontrolled dyslipidemia	1.06 (0.75–1.50)		0.78 (0.59–1.02)	

DM, diabetes mellitus; IFG, impaired fasting glucose; HTN, hypertension. Adjusted for age, income level, lifestyle factors, body mass index, DM, HTN, dyslipidemia, mental disorders, and antiplatelet use.

## Data Availability

The datasets analyzed in this study are not publicly available because of legal restrictions imposed by the Korean National Health Insurance Service (NHIS). Access to the data may be granted to qualified researchers following approval from the NHIS Big Data Steering Department and the relevant Institutional Review Board.

## Supplementary Materials

The following supporting information can be downloaded at: https://www.mdpi.com/article/10.3390/jcm15166406/s1, Table S1. Sensitivity analysis of the Parkinson’s Disease Risk in Individuals with Stroke. A combined diagnosis of secondary or atypical parkinsonism was excluded from the diagnosis of Parkinson’s disease.
