# Supplementary material for: Vascular Risk Factors and the Risk of Incident Parkinson’s Disease Among Stroke Survivors: A Nationwide Population-Based Cohort Study
Source: J Clin Med. 2026 Aug 19;15(16):6406. doi: 10.3390/jcm15166406 (PMC13513578; doi:10.3390/jcm15166406)
Supplement: Supplementary file 1 [file jcm-15-06406-s001.zip › jcm-4430363-supplementary.pdf]

Supplementary Table S1. Sensitivity analysis of the Parkinson's Disease Risk in Individuals with Stroke. A combined diagnosis of secondary or atypical parkinsonism was excluded from the diagnosis of Parkinson's disease.

		N	PD	Person-years	Incidence rate	Model 1	*p*-value	Model 2	*p*-value	Model 3	*p*-value
Sex	Male	152,270	1497	757,835.62	1.98	0.98 (0.92-1.06)		1.35 (1.23-1.47)		1.43 (1.30-1.56)	
	Female	132,157	1405	703,508.02	2.00	1.00	0.667	1.00	<.0001	1.00	<0.0001
Age (years)	40-64	131,455	589	713,560.17	0.83	1.00	<0.0001	1.00	<.0001	1.00	<0.0001
	65-	152,972	2313	747,783.46	3.09	3.73 (3.40-4.08)		3.41 (3.10-3.74)		3.32 (3.02-3.65)	
Income level	Q1	58,325	553	292,367.18	1.89	1.13 (1.00-1.29)		1.11 (0.97-1.26)		1.10 (0.97-1.25)	
	Q2	48,394	415	249,254.21	1.66	1.00	<0.0001	1.00	0.2748	1.00	0.3357
	Q3	68,546	660	355,739.21	1.86	1.12 (0.99-1.26)		1.03 (0.91-1.16)		1.03 (0.91-1.16)	
	Q4	109,162	1274	563,983.03	2.26	1.36 (1.21-1.52)		1.09 (0.97-1.22)		1.09 (0.97-1.21)	
Smoking	Never	182,844	2133	953,274.83	2.24	1.00	<0.0001	1.00	<.0001	1.00	<0.0001
	Former	63,273	531	312,749.33	1.70	0.76 (0.69-0.83)		0.76 (0.68-0.84)		0.75 (0.68-0.84)	
	Current	38,310	238	195,319.48	1.22	0.54 (0.48-0.62)		0.65 (0.57-0.76)		0.65 (0.56-0.75)	
Alcohol consumption	Non	218,170	2427	1,113,318.11	2.18	1.00	<0.0001	1.00	<.0001	1.00	0.0015
	Moderate	55,586	414	291,673.32	1.42	0.65 (0.59-0.72)		0.83 (0.74-0.92)		0.86 (0.77-0.96)	
	Heavy	10,671	61	56,352.21	1.08	0.50 (0.39-0.64)		0.67 (0.51-0.86)		0.69 (0.53-0.90)	
Physically active	No	229,076	2416	1,170,477.41	2.06	1.00	<0.0001	1.00	0.0124	1.00	0.019
	Yes	55,351	486	290,866.23	1.67	0.81 (0.74-0.89)		0.88 (0.80-0.97)		0.89 (0.81-0.98)	
Body mass index	<18.5	8311	72	35,366.43	2.04	0.99 (0.78-1.26)		0.87 (0.682-1.10)		0.85 (0.67-1.08)	
	18.5-23	91,728	939	461,596.2	2.03	1.00	0.4742	1.00	0.5629	1.00	0.3986
	23-25	74,469	783	388,650.39	2.01	0.99 (0.90-1.09)		1.03 (0.94-1.14)		1.04 (0.95-1.14)	
	25-30	98,306	1008	516,576.11	1.95	0.96 (0.88-1.05)		1.04 (0.95-1.14)		1.05 (0.96-1.15)	
	≥30	11,613	100	59,154.5	1.69	0.83 (0.68-1.02)		0.97 (0.78-1.19)		0.98(0.80-1.21)	
DM	Normal	136,363	1294	724,318.49	1.79	1.00	<0.0001	1.00	<0.0001	1.00	<0.0001
	IFG	71,223	695	362,921.15	1.92	1.07 (0.98-1.17)		1.05 (0.95-1.15)		1.04 (0.95-1.14)	

	Incident DM	11,003	113	55,073.89	2.05	1.15 (0.95-1.39)		1.13 (0.93-1.37)		1.13 (0.93-1.37)	
	Duration < 5yr.	24,506	245	127,257	1.93	1.08 (0.940-1.23)		1.05 (0.91-1.21)		1.05 (0.91-1.21)	
	Duration ≥ 5yr.	41,332	555	191,773.11	2.89	1.60 (1.45-1.77)		1.39 (1.25-1.54)		1.39 (1.25-1.53)	
HTN	Normal	58,331	504	301,512.1	1.67	1.00	<0.0001	1.00	0.4755	1.00	0.6175
	Pre-HTN	34,061	316	180,416.22	1.75	1.05 (0.91-1.21)		1.03 (0.90-1.19)		1.03 (0.90-1.19)	
	Incident HTN	6751	53	36,656.77	1.45	0.87 (0.66-1.15)		0.80 (0.60-1.06)		0.82 (0.61-1.08)	
	Controlled HTN	129,476	1392	656,211.24	2.12	1.27 (1.15-1.40)		1.03 (0.92-1.14)		1.01 (0.91-1.12)	
	Uncontrolled HTN	55,808	637	286,547.3	2.22	1.33(1.183,1.495)		1.00 (0.89-1.13)		1.00 (0.88-1.12)	
Dyslipidemia	Normal	82,598	963	446,625.06	2.16	1.00	0.0022	1.00	0.0023	1.00	0.0006
	Pre-dyslipidemia	45,976	483	257,768.85	1.87	0.87 (0.78-0.97)		0.92 (0.82-1.03)		0.92 (0.82-1.02)	
	Incident dyslipidemia	16,501	147	93,172.34	1.58	0.73 (0.62-0.87)		0.78 (0.65-0.93)		0.78 (0.66-0.93)	
	Controlled dyslipidemia	131,435	1232	620,510.19	1.99	0.91 (0.84-0.99)		0.85 (0.78-0.93)		0.84 (0.77-0.91)	
	Uncontrolled dyslipidemia	7917	77	43,267.2	1.78	0.83 (0.66-1.04)		0.86 (0.68-1.09)		0.81 (0.64-1.02)	

DM, diabetes mellitus; IFG, impaired fasting glucose; HTN, hypertension

Incidence rate is the incidence of mortality per 1000 person-years

Model 1: unadjusted

Model 2: adjusted for age, sex, Income level, lifestyle factors, BMI, DM, HTN, and dyslipidemia

Model 3: adjusted for age, sex, Income level, lifestyle factors, BMI, DM, HTN, dyslipidemia, mental disorders, and antiplatelet use
